# Genetic Variants and Phenotypic Characteristics of *Salmonella* Typhimurium-Resistant Mutants after Exposure to Carvacrol

**DOI:** 10.3390/microorganisms8060937

**Published:** 2020-06-22

**Authors:** Daniel Berdejo, Natalia Merino, Elisa Pagán, Diego García-Gonzalo, Rafael Pagán

**Affiliations:** Departamento de Producción Animal y Ciencia de los Alimentos, Facultad de Veterinaria, Instituto Agroalimentario de Aragón-IA2 (Universidad de Zaragoza-CITA), 50013 Zaragoza, Spain; berdejo@unizar.es (D.B.); merino@unizar.es (N.M.); epagan@unizar.es (E.P.); Diego.Garcia@unizar.es (D.G.-G.)

**Keywords:** antimicrobial resistance, evolution assays, carvacrol, *Salmonella* Typhimurium, genotypic resistance, antibiotics, whole-genome sequencing, resistant mutants

## Abstract

The emergence of antimicrobial resistance has raised questions about the safety of essential oils and their individual constituents as food preservatives and as disinfection agents. Further research is required to understand how and under what conditions stable genotypic resistance might occur in food pathogens. Evolution experiments on *Salmonella* Typhimurium cyclically exposed to sublethal and lethal doses of carvacrol permitted the isolation of SeSCar and SeLCar strains, respectively. Both evolved strains showed a significant increase in carvacrol resistance, assessed by minimum inhibitory and bactericidal concentrations, the study of growth kinetics in the presence of carvacrol, and the evaluation of survival under lethal conditions. Moreover, antibiotic susceptibility tests revealed a development of SeLCar resistance to a wide range of antibiotics. Whole genome sequencing allowed the identification of single nucleotide variations in transcriptional regulators of oxidative stress-response: *yfhP* in SeSCar and *soxR* in SeLCar, which could be responsible for the increased resistance by improving the response to carvacrol and preventing its accumulation inside the cell. This study demonstrates the emergence of S. Typhimurium-resistant mutants against carvacrol, which might pose a risk to food safety and should therefore be considered in the design of food preservation strategies, or of cleaning and disinfection treatments.

## 1. Introduction

One of the greatest challenges to global health over the last two decades has been the emergence of antimicrobial resistance (AMR) and its spread in the environment [1,2]. This concern has shifted from clinical setups to further areas, raising questions about the safety of natural preservatives, such as essential oils (EOs) and individual constituents (ICs), as food preservatives or as cleaning and disinfection agents. Carvacrol is an IC mainly extracted from EO of *Origanum vulgare*, *Thymus vulgaris* and *Thymbra capitata* [3,4], generally recognized as safe (GRAS) by the U.S. Food and Drug Administration [5]. This monoterpenoid phenol has demonstrated outstanding antimicrobial properties [6,7,8] and is therefore regarded as one of the most promising ICs as a food preservative or as a cleaning and disinfection agent [9].

Since high antioxidant activity of EOs and ICs at low doses [10] would normally reduce the mutation frequency of treated bacteria [11,12], it has been commonly accepted that these compounds do not induce mutations that could lead to AMR [13]. In this regard, previous studies of evolution assays in *Salmonella* did not observe increased resistance after exposure to subinhibitory amounts of *Origanum vulgare* and carvacrol [14]. However, Chueca, Berdejo, Gomes-Neto, Pagán and García-Gonzalo [11] and Berdejo, et al. [15] described for the first time the emergence of mutations in bacterial populations after prolonged cyclic exposure to subinhibitory doses of these compounds, thereby resulting in increased bacterial resistance. The identification of the genetic modifications in those resistant mutants led to a better understanding of the bacterial response against ICs and, consequently, of their mechanisms of action [15,16]. Interesting results were obtained in carvacrol evolution treatments in *Escherichia coli*: a mutation in the *soxR* gene was marked as responsible for a significant increase in resistance not only to carvacrol, but also to other ICs and even to antibiotics [11,16]. Moreover, a recent study reported the emergence of resistant strains of *E. coli* O23:H52 against carvacrol and oregano by cyclic exposure to subinhibitory doses [17]. In this regard, it is unknown whether the increased resistance observed in *E. coli* [11] or in *Staphylococcus aureus* [15] might also occur in one of the food pathogens most involved in food outbreaks, *Salmonella* spp., and whether the mutagenesis might follow a general pattern, or rather a specific one, as a function of the type of microorganism, of the bacteriostatic agent, or of treatment conditions.

Moreover, several studies have isolated resistant mutants from the tail of survival curves after cyclic lethal treatments with physical agents, such as heat [18] and high hydrostatic pressure [19], or with chemical agents, such as antibiotics [20]. However, it is unknown whether the application of lethal doses of EOs or ICs might favor the emergence of resistant mutants, as observed at sublethal doses, thus posing a risk to food safety.

For these reasons, further research is still needed in order to describe the occurrence of AMR under sublethal or lethal carvacrol concentrations, paving the way for further in-depth exploration of carvacrol’s mechanisms of action. This knowledge would contribute towards enhancing the antimicrobial properties of carvacrol as a single agent or in combined processes [15] with other antimicrobial agents [21] or with physical treatments [22].

This study therefore seeks (a) to isolate mutant-resistant strains of *S. enterica* Typhimurium under two different protocols of carvacrol evolution assays: cyclic exposure to prolonged sublethal treatments, and cyclic exposure to short lethal treatments; (b) to describe the resistance of the isolated strains against carvacrol and antibiotics; (c) to identify the mutations involved in the observed bacterial resistance. 

## 2. Materials and Methods 

### 2.1. Microorganisms and Growth Conditions

*Salmonella enterica* subsp. *enterica* serovar Typhimurium LT2 (SeWT) was provided by the Spanish Type Culture Collection (CECT 722). Isolated in the 1940s, it is one of the principal strains used in cellular and molecular biology studies of *Salmonella* since its genome was completely sequenced in 2001 [23]. For this reason, we selected this strain to carry out our study of genetic evolution under selective pressure from carvacrol.

Throughout this investigation, the strain was kept in cryovials at −80 °C with glycerol (20% v/v), from which plates of tryptone soya agar (Oxoid, Basingstoke, England) with 0.6% yeast extract (Oxoid; TSAYE) were prepared on a weekly basis. To prepare the working bacterial cultures, test tubes containing 5 mL of tryptone soya broth (Oxoid,) with 0.6% yeast extract (TBSYE) were inoculated with one colony and then incubated aerobically on an orbital shaker (130 rpm; Heidolph Vibramax 100, Schwaback, Germany) for 12 h at 37 °C (Incubig, Selecta, Barcelona, Spain). Subsequently, flasks containing 10 mL of fresh TSBYE were inoculated with 2 µL of the resulting subculture to achieve an initial concentration of 10^6^ colony forming units per mL (CFU/mL), and incubated for 24 h at 37 °C and 130 rpm until the stationary growth phase was reached (5 × 10^9^ CFU/mL approximately). The bacterial concentration of the cultures was verified by spreading them on TSAYE plates. We applied the same protocol to obtain the working bacterial cultures of the isolated strains that resulted from the evolution experiments with carvacrol in this study.

### 2.2. Minimum Inhibitory Concentration (MIC) and Minimum Bactericidal Concentration (MBC)

The minimum inhibitory concentration determination was performed by inoculating selected strains in test tubes with 5 mL of mueller hinton broth cation (Sigma-Aldrich; MHB) adjusted to achieve an initial concentration of 5 × 10^5^ CFU/mL in the presence of different concentrations of carvacrol: from 50 up to 500 µL/L, and incubated at 37 °C for 24 h and 130 rpm. Once the tubes were incubated, MIC was determined as the lowest concentration of the antimicrobial compound that was capable of avoiding bacterial growth. To objectively determine bacterial growth, the optical density was read at 595 nm (OD_595_) using a microplate reader (Genios, Tecan, Männedorf, Switzerland). An amount of 10% of the OD_595_ measure of the positive control was established as the lower limit to consider that bacterial strain was grown [24]. Following the method described by Friedman et al., [25], a vigorous shaking by vortex (Genius 3, Ika, Königswinter, Germany) was used to prepare carvacrol dispersions in MHB, avoiding the use of solvents for their possible detriment in the antibacterial activity. Positive control tubes with 5 mL MHB inoculated at 5 × 10^5^ CFU/mL without ICs, and negative control tubes with 5 mL MHB inoculated at the same concentration with 1000 µL/L of carvacrol, were also prepared in every experiment. This protocol was adapted from standard methods for antimicrobial susceptibility tests [26].

The minimum bactericidal concentration (MBC) of carvacrol was evaluated in parallel to the MIC test. From the test tubes employed in the MIC determination after incubation, 100 µL aliquot of each tube was spread onto mueller hinton agar cation-adjusted (Sigma-Aldrich; MHA) plates and incubated at 37 °C for 24 h. Colonies were counted and the lowest concentration of carvacrol that killed ≥ 99.9% of the initial bacterial concentration (5 × 10^5^ CFU/mL) was defined as the MBC end point [27]. The same positive and negative controls as the MIC test were employed in this experiment. The MBC of evolved strains were compared to that of SeWT to assess the increased resistance to carvacrol.

### 2.3. Carvacrol Evolution Assays

**Figure 1:**
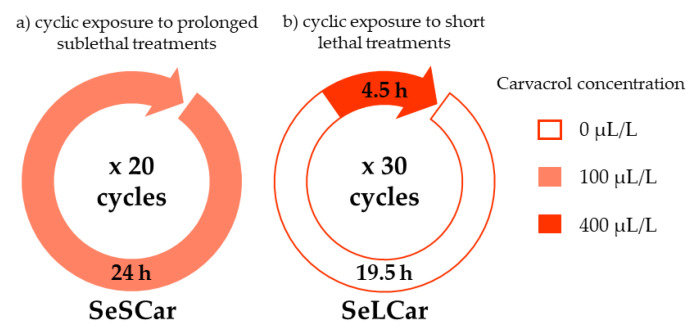
Schematic for the experimental protocols of carvacrol evolution assays: (**a**) cyclic exposure to prolonged sublethal treatments (SeSCar) and (**b**) cyclic exposure to short lethal treatments (SeLCar).

The use of ICs in food preservation can lead either to the inhibition of bacterial growth or to bacterial inactivation, depending on IC concentration. Then, to obtain resistant *Salmonella* strains against carvacrol, two different protocols were followed in order to simulate bacteriostatic and bactericidal conditions: (a) cyclic exposure to prolonged sublethal treatments, and (b) cyclic exposure to short lethal treatments (Figure 1).

**Figure 2:**
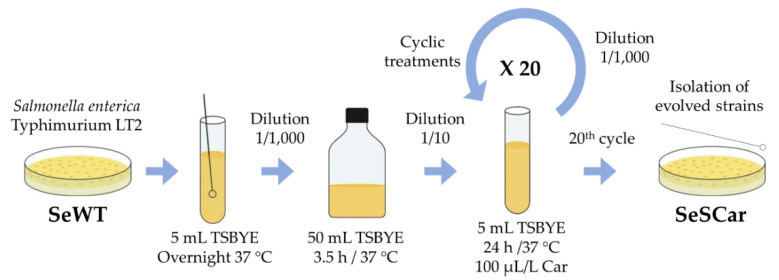
Diagram of evolution assay by prolonged sublethal treatments.

**Figure 3:**
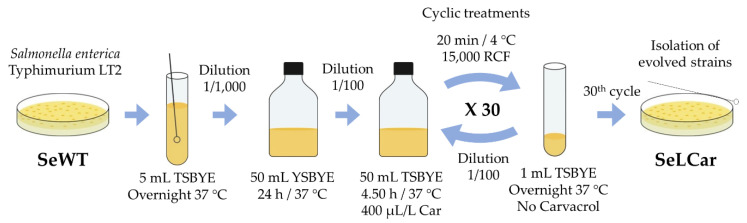
Diagram of evolution assay by short lethal treatments.

(a) The first protocol was based on the isolation of strains by prolonged exposure to a subinhibitory concentration of carvacrol during the growth phase of bacteria (Figure 2)*. Salmonella* wild-type strain (SeWT) was grown on TSAYE plates for 24 h at 37 °C. A single colony was inoculated in 5 mL TSBYE and incubated under agitation for 12 h at 37 °C. This preculture was diluted 1:1000 into 50 mL TSBYE and incubated for 3.5 h to obtain an exponential phase culture. From this culture, 5 mL TSBYE were inoculated at an initial bacterial concentration of 10^6^ CFU/mL in the presence of 100 µL/L of carvacrol (1/2 of MIC for SeWT). The bacterial concentration of the cultures was verified by spreading them on TSAYE plates. This bacterial suspension was incubated 24 h/37 °C/130 rpm and, once the stationary phase was reached, the same step was repeated: the previous culture was diluted (10^6^ CFU/mL) in 5 mL TSBYE with 100 µL/L of carvacrol (≥ 98%; Sigma-Aldrich) and incubated 24 h/37 °C/130 rpm. This procedure was repeated 20 times. After the 20^th^ step, an aliquot was diluted in phosphate-buffered saline (Sigma-Aldrich, Steinheim, Westphalia, Germany; PBS) and spread on TSAYE plates (without carvacrol). After the incubation period, 5 colonies (SeSCar_1-5_) were randomly selected to carry out phenotypic and genotypic characterization. This methodology was adapted from Kohanski, et al. [24] and Andersson and Hughes [28]. This approach mimes the use of carvacrol together with other natural substances for preventation purposes.

(b) The second protocol was based on the isolation of strains by recovering surviving cells after short-term lethal treatments with carvacrol (Figure 3). For this purpose, a stationary phase culture of SeWT was diluted 1:100 in 50 mL fresh TSBYE with 400 µL/L of carvacrol (2 × MIC for SeWT) for 4.50 h at 37 °C. Subsequently, treated cells were centrifuged for 20 min at 15,000 RCF, washed twice with TSBYE, resuspended in 1 mL TSBYE and incubated overnight at 37 °C. This procedure was repeated 30 times. After the 30th step, an aliquot was diluted in PBS and spread on TSAYE plates (without carvacrol), from which 5 colonies (SeLCar_1-5_) were randomly selected to carry out phenotypic and genotypic characterization. This methodology was adapted from Levin-Reisman, Ronin, Gefen, Braniss, Shoresh and Balaban [20]. This approach simulates a single treatment after a *Salmonella* contamination.

Once the 5 strains isolated by each evolution assay, SeSCar_1-5_ and SeLCar_1-5_, were obtained, the first approach to evaluate their resistance was to determine the MIC and the MBC of carvacrol and to compare with those of the SeWT.

### 2.4. Growth Curves in Presence of Carvacrol

In order to more deeply study the behavior of the isolated strains against carvacrol, the growth kinetics of SeWT and of evolved strains were evaluated in TSBYE at different concentrations of carvacrol.

First, carvacrol was added at different concentrations in tubes with 5 mL of TSBYE. Based on the results obtained in the MIC assay, the concentration range of carvacrol used was 0–150 μL/L for SeWT, 0–250 μL/L for SeSCar and 0–350 μL/L for SeLCar. Due to the hydrophobicity of carvacrol, it was necessary to apply vigorous agitation in the vortex to get a uniform suspension. Once the IC was added, test tubes were inoculated with the microbial culture at an initial concentration of 5 × 10^5^ CFU/mL and incubated at 37 °C and 130 rpm for 24 h. Every hour, OD_595_ of the test tubes was measured by a microplate reader. The experiment was prolonged for more than 24 h at high carvacrol concentrations until reaching the stationary growth phase. A positive control (without antimicrobial added) and a negative control (without microbial culture added) were incorporated in all the assays. The values of OD_595_ obtained during the experiment was subtracted from the initial OD_595_ (at time 0), corresponding to the absorbance caused by the growth medium. Bacterial growth curves based on OD_595_ of SeWT, SeSCar, and SeLCar were graphically displayed and modelled by a modified Gompertz equation [29]:

(1) $$y=A\exp\left\{-exp\left[\;\left({µ}_{m}\;e\;/A\;\right)\left(\lambda -t\right)+1\;\right]\right\}$$

where y: OD_595_; t: time (h); *A*: maximum value reached (OD_595_ max); *µ_m_*: maximum specific growth rate (h^−1^); *λ*: lag time (h).

A least-squares adjustment was carried out to build the model and obtain *A*, *µm* and *λ* values using the GraphPrism® program (GraphPad Software, Inc., San Diego, CA, USA). The adjustment’s goodness of fit was evaluated using standard error, *R^2^* and *R^2^* adjusted values, and the root mean square error (*RMSE*).

### 2.5. Survival Curves in Presence of Carvacrol

The resistance of SeWT and of the evolved strains against carvacrol was also evaluated with lethal treatments. In these cases, the treatment medium we used was citrate–phosphate buffer or “McIlvaine buffer”, prepared from citric acid monohydrate (Panreac) and disodium hydrogen phosphate (Panreac), adjusted to pH 4.0 and pH 7.0. These pH values were chosen as representative of neutral and acid conditions. The treatment was carried out in 10 mL McIlvaine buffer previously tempered at 25 °C, to which carvacrol was added at a concentration of 150 µL/L and then vigorously agitated to obtain a homogeneous dispersion of the IC. This concentration was selected based on preliminary experiments using 100–300 µL/L of carvacrol against SeWT strain (results not shown), in order to apply a treatment that would achieve 5 log_10_ cycles of inactivation and whose inactivation kinetics would permit comparison with the resistance of the evolved strains. Once carvacrol was added, stationary phase culture was centrifuged for 5 min at 6000 RCF in a microcentrifuge (Mini Spin, Eppendorf, Hamburg, Germany) and resuspended in the treatment medium. Test tubes were then inoculated at 10^7^ CFU/mL, thus initiating the lethal carvacrol treatment. Total treatment time was set to 30 min, during which aliquots were obtained every 5 min. These samples were diluted in PBS and subsequently spread on TSAYE plates. After plate incubation (24 h/ 37 °C), the count of survival cells was carried out in an automatic plate counter by image analysis (Analytical Measuring Systems, Protos, Cambridge, United Kingdom). Once survival curves of SeWT and evolved strains were obtained, inactivation kinetics were compared in order to evaluate the increase in resistance of SeSCar and SeLCar against carvacrol.

### 2.6. Antibiotic Susceptibility Test

Agar disk diffusion assay was used to test antimicrobial susceptibility according to CLSI [27,30]. First, bacterial suspension was spread on MHA plates and, after 5 min at room temperature, blank disks (Ø: 6.0 mm) (Thermo Scientific™ Oxoid™ Anti-microbial Susceptibility Disk Dispenser, ST6090, Waltham, MA, USA) were placed on the surface of plates and individually impregnated with 10 μL of each antibiotic: 30 µg kanamycin sulfate, 30 µg tetracycline, 30 µg chloramphenicol, 400 µg nalidixic acid sodium, 50 µg rifampicin, 20 µg norfloxacin, 250 µg novobiocin sodium, 10 µg trimethoprim, 10 µg ampicillin, and 150 µg cephalexin (Sigma-Aldrich). These plates were incubated at 37° C for 18–24 h, after which the diameters of the resulting inhibition zones were measured (paper disks included). We selected the range of antibiotics in order to cover different cellular targets that could be related to carvacrol resistance.

### 2.7. Whole Genome Sequencing (WGS) and Identification of Genetic Variations

Genomic DNA (gDNA) was extracted using a gDNA kit (DNeasy kit, Qiagen, Hilden, Germany) for extraction and purification of SeWT and the evolved strains. Illumina technology was used to carry out whole genome sequencing (WGS) on NextSeq equipment at mid-output flow, with a total of 2 × 150 cycles (Illumina; Fasteris, SA, Geneva, Switzerland). Subsequently, quality control was performed with FastQC software (https://www.bioinformatics.babraham.ac.uk/projects/fastqc/) evaluating reading quality (Q_30_), sequence length, presence of adapters, and overrepresented and duplicated sequences. The quality-control-filtered paired-end reads were mapped on the reference genome sequence (National Center for Biotechnology Information; NCBI accession: NC_003197.2): *Salmonella enterica* subsp. *enterica* serovar Typhimurium str. LT2, complete genome [23], using a Burrows–Wheeler Alignment (BWA) Tool [31] and Samtools software [32] (sources: http://bio-bwa.sourceforge.net/ and http://www.htslib.org/). A raw-coverage 150-fold depth was achieved for the three strains. Then, Samtools was applied to remove potential PCR duplicates according to reading positions on the reference genome; the resulting BAM files were then further processed using LoFreq-Star (source: http://csb5.github.io/lofreq/) to correct mapping errors and insert the quality values. Finally, single nucleotide variants (SNVs) and short insertion and deletions (InDels) were detected using LoFreq-Star, and toolbox snpEff (source: http://snpeff.sourceforge.net/) was employed to identify involved genes and to predict functional effect variations [33]. Coverage was further analysed by the Integrative Genomics Viewer (IGV; Broad Institute, source: https://software.broadinstitute.org/software/igv/) in order to find structural variations (SVs). Although mapping was carried out against the reference genome, SNVs, InDels, and SVs were identified between SeWT and isolated strains to ascertain the kind of mutations that had occurred during the evolution treatments. Finally, specific primers (Table S1) were designed with the “Primer designing tool” of NCBI to carry out PCR amplifications, as well as Sanger sequencings to verify the mutations detected in the WGS. Sanger sequencing reads were aligned and compared using the software Bioedit (http://www.mbio.ncsu.edu/BioEdit/bioedit.html). The resulting genome sequences were deposited in the Sequence Read Archive (SRA) of NCBI (BioProject ID: PRJNA634825). The accession numbers of the samples are SAMN15009803 (SeWT), SAMN15009804 (SeSCar), SAMN15009805 (SeLCar). Additionally, Table S2 summarizes the genomic background of *S.* Typhimurium LT2.

### 2.8. Statistical Analysis

All phenotypic characterization results were obtained from at least 3 independent experiments carried out on different working days with different bacterial cultures. MIC and MBC data correspond to the results obtained from 5 different assays. Growth curve parameters, lethal treatment graphics, and antibiotic susceptibility tests are displayed as the mean ± standard deviation, using the GraphPrism® program. Data were analyzed and submitted to comparison of averages using analysis of variance (ANOVA), followed by post-hoc Tukey test and t-tests with GraphPrism®, and differences were considered significant if *p* ≤ 0.05.

## 3. Results and Discussion

### 3.1. Isolation of Resistant Strains Obtained by Selective Pressure of Carvacrol

Two different protocols were followed to obtain resistant *Salmonella* strains against carvacrol: (a) cyclic exposure to prolonged sublethal treatments, and (b) cyclic exposure to short lethal treatments. From each evolution experiment, five colonies, (a) SeSCar_1-5_ and (b) SeLCar_1-5_, were randomly isolated after 20 and 30 cycles, respectively. Subsequently, phenotypic and genotypic characterization were performed to determine whether the carvacrol evolution assays allowed for the emergence of stable resistant bacterial strains.

The resistance of SeSCar_1-5_ and SeLCar_1-5_ against carvacrol was determined by assaying the minimum inhibitory concentration (MIC) and the minimum bactericidal concentration (MBC) (Table 1). The results of the evolved strains were compared with those of SeWT in order to assess increased resistance to carvacrol.

On the one hand, the bacteriostatic effect of carvacrol on *S. enterica* strains (SeWT, SeSCar_1-5_ and SeLCar_1-5_.) was evaluated by MIC determination (Table 1). The data of the five isolated colonies from the same evolution experiment were grouped in the same row, since the MIC results displayed the same values (*p* < 0.05). The MIC results demonstrated the strong antibacterial activity of carvacrol against *S.* Typhimurium. Similar MIC values have been obtained in other studies against *Salmonella* strains. The MIC determined by Mith, et al. [34] was 125 µL/L for *S.* Typhimurium CDC 6516-60 (ATCC 14028), and 188 µL/L for *S.* Typhimurium S0584 (isolated from pig carcass). Lu and Wu [35] obtained a MIC of 205 µL/L carvacrol against another *S*. Typhimurium strain.

As detailed in Table 1, both isolated mutants showed an increase in the MIC of carvacrol, from 200 µL/L against SeWT to 300 µL/L against SeSCar_1-5_, and to 400 µL/L against SeLCar_1-5_. This corresponds to 50% and 100% increased resistance after the carvacrol evolution treatments. Chueca, Berdejo, Gomes-Neto, Pagán and García-Gonzalo [11] and Berdejo, Chueca, Pagan, Renzoni, Kelley, Pagan and Garcia-Gonzalo [15] also observed an increase in resistance to carvacrol in the strains evolved by exposure to subinhibitory doses: a 300% increase of the MIC against *Escherichia coli* MG1655, and 50% against *Staphylococcus aureus* USA300, respectively. The MIC for SeSCar_1-5_, evolved in the presence of subinhibitory doses, and was lower than that of SeLCar_1-5_ evolved by lethal doses. However, there are no previous reports on the MIC determination of carvacrol for strains evolved by cyclic exposure to lethal doses.

The bactericidal effect of carvacrol was explored by MBC determination. As in the MIC test, the MBC values for the evolved strains obtained with the same protocol were identical (*p* < 0.05) and are consequently grouped in Table 1. The MBCs of carvacrol were the same as the MIC values for SeWT (200 µL/L) and for the evolved strains: 300 µL/L for SeSCar_1-5_ and 400 µL/L for SeLCar_1-5_. Similar MIC and MBC values have been associated with the strong bactericidal activity of carvacrol even at low concentrations. For instance, no differences between MIC and MBC of carvacrol were detected against *Escherichia coli* O157:H7; both concentrations reached values of 200 µL/L [22]. However, Lu and Wu [35] and Mith et al. [34] observed different MBC and MIC values of carvacrol for each tested *S.* Typhimurium strain: between 200 and 400 µL/L and between 125 and 375 µL/L, respectively. Therefore, divergence among MIC and MBC values would be more due to strain-to-strain variation than to bactericidal activity of the antimicrobial compound.

Regarding the comparison of evolved strains with SeWT, MBC increased by 50% in SeSCar_1-5_ and 100% in SeLCar_1-5_. As shown by the MIC test, SeLCar exhibited a greater resistance than SeSCar: this could be due to the protocol applied in the corresponding evolution experiment, which applied a bactericidal concentration (400 µL/L). Nevertheless, there are no previous studies on the MBC assessment of carvacrol for evolved strains of any microorganism. These results reveal that evolved strains show a higher resistance to carvacrol, and resistance varies as a function of the method of evolution. In this regard, lethal treatments seem to lead to the emergence of more resistant strains than using sublethal doses.

The protocol we followed in the evolution assays with sublethal doses of carvacrol had been employed in previous studies on *E. coli* [11] and *S. aureus* [15]. In both studies, the evolved strains revealed increased resistance to carvacrol, and even cross-resistance to other ICs and antibiotics. In contrast, Gomes-Neto, et al. [36] did not observe the emergence of resistant strains of *S.* Typhimurium against *Rosmarinus officinalis* L. EO and 1,8-cineole in an evolution assay with subinhibitory doses but increasing the concentration in the course of the experiment. Regarding evolution assays with lethal treatments of EOs and ICs, to the best of our knowledge, no previous studies have evaluated the appearance of AMR: neither in *Salmonella* spp., nor in any other microorganism against these natural compounds. MIC and MBC results revealed that the resistance of all the colonies coming from the same evolution lineage displayed the same degree of resistance to carvacrol. These results suggest that all isolated colonies were identical, and that the bacterial cultures obtained from the evolution treatments were probably homogeneous. We therefore pursued the remainder of our research with one of the five strains selected from each of the evolution protocols: SeSCar (obtained by cyclic exposure to prolonged sublethal doses) and SeLCar (obtained by cyclic exposure to short lethal treatments).

### 3.2. Growth Kinetics under Carvacrol Stress

**Figure 4:**
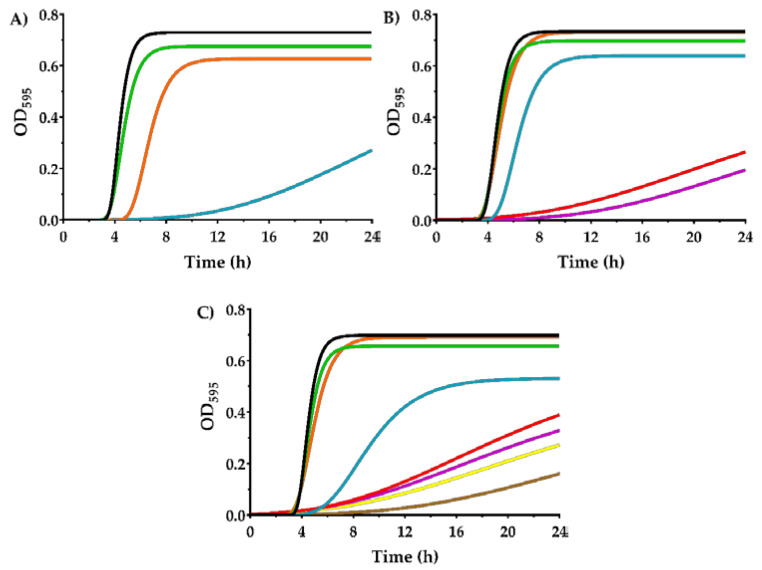
Growth curves of *Salmonella enterica* subsp. *enterica* serovar Typhimurium LT2 wild type (**A**); SeWT) and evolved strains: SeSCar (**B**); by cyclic exposure to prolonged sublethal treatments of carvacrol) and SeLCar (**C**); by cyclic exposure to short lethal treatments of carvacrol), in the absence (▬) and presence of 50 (▬), 100 (▬), 150 (▬), 200 (▬), 250 (▬), 300 (▬) and 350 µL/L (▬) of carvacrol, modelled using the modified Gompertz equation (Equation (1)).

The growth kinetics in the presence of carvacrol were evaluated in order to describe in depth the behavior of the evolved strains against that IC. Figure 4 displays the growth curves of SeWT, SeSCar, and SeLCar modelled by modified Gompertz equation (Equation (1)) in the presence of varying concentrations of carvacrol (from 0 to 350 µL/L). The standard error, *R*^2^ and *R*^2^ adjusted values and the root mean square error (*RMSE*) supported a good least-squares adjustment (Table S3). In agreement with the MIC results, concentrations higher than 150 µL/L did not allow the growth of SeWT (Figure 4A); neither did those higher than 250 µL/L for SeSCar (Figure 4B), nor those higher than 350 µL/L for SeLCar (Figure 4C). 

As can be observed in Figure 4, all strains showed an extended lag phase and a decrease in the maximum growth rate as the concentration of carvacrol was increased. However, this effect was more pronounced for SeWT than for the evolved strains. For instance, SeWT could not reach the stationary growth phase at 24 h in the presence of 150 µL/L carvacrol, whereas the evolved strains reached the stationary phase under the same conditions before 20 h. The parameters of the modified Gompertz equation: *A* (maximum OD_595_), *µ_m_* (maximum specific growth rate) and *λ* (lag time), for the three strains and under all the conditions tested, are provided in Table 2.

As can be seen, maximum OD_595_ slightly decreased as the concentration of carvacrol in the growth medium increased. However, no statistically significant differences (*p* ≥ 0.05) were observed between SeWT and the evolved strains at carvacrol concentrations below the MIC of SeWT (< 200 µL/L). Regarding the maximum growth rate, a strong decrease thereof was noted in the three strains as the carvacrol concentration increased. This growth parameter also showed significant differences (*p* < 0.05) between SeWT and the evolved strains when carvacrol was added to the medium: at 150 µL/L, the maximum growth rate of SeWT was 0.024 OD_595_/h, while SeSCar and SeLCar reached values of 0.262 and 0.088 OD_595_/h, respectively. The lag phase was prolonged by the presence of carvacrol in the growth medium for the three strains (*p* < 0.05), but this effect was more pronounced in SeWT. The lag time of SeWT was longer than those of the evolved strains at all tested carvacrol concentrations above 50 µL/L. For instance, the lag time in SeWT at 150 µL/L was 12.8 h, which was 7 h and 6 h longer than in SeSCar and SeLCar, respectively.

Comparing the evolved strains, even though the MIC and MBC results revealed a greater resistance of SeLCar, the growth curves at low carvacrol concentrations (100–150 µL/L) displayed a higher growth rate of SeSCar. This improved adaptation of SeSCar to low doses of carvacrol is probably the consequence of the protocol followed in the evolution experiments, since the concentration used to obtain SeSCar was 100 µL/L.

The effect of the presence of EOs and ICs on bacterial growth has been previously studied. According to Braschi, et al. [37], a slower growth rate and a higher lag phase was observed in *Listeria monocytogenes* as the concentration of carvacrol in the medium increased. Similar results were obtained by Melo, et al. [38] in *E. coli* and *S. aureus* against *Ocimum gratissimum* L. EO: a reduced growth rate and a lag phase delay were observed at higher EO concentrations. Nevertheless, to the best of our knowledge, no previous studies have shown the influence of the presence of any EO or IC on the growth parameters of resistant mutants obtained by cyclic exposure to the same inhibitory agents.

The high growth rate and the short lag phase observed in the growth curve of SeSCar at 100 µL/L of carvacrol, compared to that of SeWT, reveals that the evolved strain could have emerged in the evolution assays by subinhibitory doses. In addition, the growth kinetics of the evolved strains compared with SeWT support not only the possibility of the emergence of resistant strains, but also a better growth fitness in the presence of carvacrol.

### 3.3. Evaluation of Cell Survival against Carvacrol 

**Figure 5:**
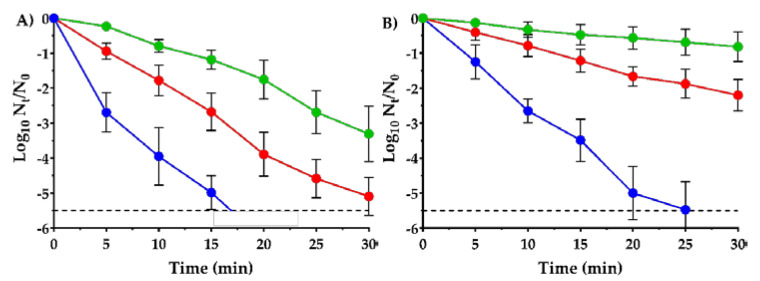
Survival curves of *Salmonella enterica* subsp. *enterica* serovar Typhimurium LT2 wild type (●); SeWT) and evolved strains: SeSCar (●); by cyclic exposure to prolonged sublethal treatments of carvacrol) and SeLCar (●); by cyclic exposure to short lethal treatments of carvacrol), after 150 µL/L carvacrol treatment at pH 4.0 (**a**) and pH 7.0 (**b**). Data are means ± standard deviations (error bars) obtained from at least 3 independent experiments. The dashed line represents the detection limit (−5.5 log_10_ N_t_/N_0_).

Lethal treatments at 150 µL/L of carvacrol were applied to SeWT and the evolved strains at pH 4.0 and pH 7.0 (Figure 5), within the normal pH range of food.

As can be seen in Figure 5, only 150 µL/L of carvacrol were needed to reduce 5 log_10_ cycles of SeWT in 15 min at acidic pH, and in 20 min at neutral pH. This assay corroborates the strong bactericidal properties of carvacrol at low concentrations against *S.* Typhimurium, even after short treatments. Previous studies have also observed a great effectiveness of carvacrol against *S.* Typhimurium strains: Chung, Cho and Rhee [21] reported a reduction of 2 log_10_ cycles in 5 min of treatment at 2 mM concentration of carvacrol (approx. 300 µL/L). A greater inactivation was reached by Mattson, Johny, Amalaradjou, More, Schreiber, Patel and Venkitanarayanan [8]: 7 log_10_ cycles of reduction of *S.* Typhimurium was achieved in just 1 min of treatment but using higher concentrations, e.g., 2500 µL/L of carvacrol.

Additionally, comparing the survival curves at pH 4.0 (Figure 5A) with those at pH 7.0 (Figure 5B), a greater bactericidal activity of carvacrol was observed in acid medium than in neutral medium against all three strains. For instance, 5.5 log_10_ cycles of reduction of SeWT were reached at 17 min in acid pH, whereas up to 25 min were required in neutral pH. The hurdle effect between carvacrol and acid pH was already observed in two previous studies against *E. coli* [7,22]. In addition, EOs are more hydrophobic at acid pH, and therefore might interact better with the lipid bilayer of the cell membrane, thereby achieving cell injury or inactivation [39].

At acid pH, the evolved strains SeSCar and SeLCar showed a higher survival to the lethal treatment with carvacrol than SeWT. While over 5.5 log_10_ cycles of SeWT inactivation were achieved after 20 min of treatment, only 1.8 log_10_ cycles of SeSCar and 3.9 log_10_ cycles of SeLCar were inactivated within the same period. Similar results were obtained at neutral pH (Figure 5B): a greater resistance of SeSCar and SeLCar was also observed after lethal treatments at pH 7.0 compared to SeWT. For instance, whereas only 0.8 log_10_ cycles of SeSCar and 2.2 log_10_ cycles of SeLCar were inactivated after 30 min of treatment, 5.5 log_10_ cycles of reduction of SeWT were achieved within the same period. Comparing the evolved strains with one another, SeSCar displayed a greater survival rate than SeLCar at both pHs: after 30 min of treatment, SeLCar was inactivated 1.8 log_10_ cycles more than SeScar at acidic pH, and 1.4 log_10_ cycles more at neutral pH. A previous study by Chueca, Berdejo, Gomes-Neto, Pagán and García-Gonzalo [11] showed that evolution experiments on *E. coli* with subinhibitory doses of carvacrol resulted in strains that were even resistant to lethal treatments. A subsequent study with these strains revealed that combined treatments of carvacrol and heat were required to achieve comparable cell inactivation of mutant strains at low treatment intensities [40]. Strains of *S. aureus* likewise increased their resistance to lethal carvacrol treatments due to the improved bacterial repair systems in mutant strains isolated in evolution experiments at subinhibitory doses [15].

Similarly to the MIC, MBC and growth curve results discussed previously, the survival curves confirm the emergence of resistant strains of *S.* Typhimurium to carvacrol, not only after cyclic exposure to prolonged treatments at low doses, to which the bacteria can adapt, but also after cyclic exposure to short lethal treatments. However, contrary to what was expected considering the evolution protocols we followed, SeSCar, obtained under the presence of a subinhibitory concentration of carvacrol, showed a greater survival rate than SeLCar under lethal treatments of carvacrol at both acid and neutral pH. 

This is the first study that proves that the application of carvacrol, either under prolonged periods at low doses or with short repeated lethal treatments, allows the emergence of resistant strains. Previous researchers have also observed an increased resistance after a prolonged exposure to subinhibitory doses of carvacrol in other bacteria, but not after lethal treatments. In addition, the development of AMR in *Salmonella* spp. against natural antimicrobials had not been previously reported. These resistant mutants could grow at inhibitory doses or survive lethal carvacrol treatments, which would compromise food safety. In this regard, the emergence of resistant strains should be taken into account in the design of food preservation strategies to ensure consumer health.

### 3.4. Study of Antibiotic Susceptibility

As the last step in our phenotypic characterization, an antibiotic susceptibility test was conducted in order to ascertain whether any cross resistance with antibiotics could be detected. First, a preliminary control experiment was performed under the conditions shown in “Table 2A, zone diameter interpretative standards for *Enterobacteriaceae*“of CLSI [30] to assess the antibiotic resistance of SeWT (data not shown). The results demonstrated that antibiotic inhibition halos of SeWT were within the “intermediate range” according to CLSI [30], except against tetracycline and chloramphenicol, where the inhibition was higher (“susceptible”). Antibiotic concentration was subsequently increased to achieve larger halos (> 15.0 mm), and thus to increase analysis sensitivity (except for novobiocin and ampicillin, which were limited by their solubility).

Table 3 reports the inhibition halos of SeWT and of the evolved strains (Ø: 6.0 mm, included) against kanamycin, tetracycline, chloramphenicol, nalidixic acid, rifampicin, norfloxacin, novobiocin, trimethoprim, ampicillin, and cephalexin. No significant differences (*p* ≥ 0.05) in antibiotic resistance were observed between SeWT and SeSCar by agar disk diffusion assay. However, it must be noted that Chueca, Renzoni, Berdejo, Pagan, Kelley and Garcia-Gonzalo [16] found an increased antibiotic resistance in mutant strains of *E. coli* evolved with subinhibitory doses of carvacrol, citral, and limonene oxide. In contrast, against all antibiotics tested except kanamycin and cephalexin, SeLCar exhibited an increased resistance compared to the SeWT strain (*p* < 0.05). In this regard, mutations in SeLCar are likely to trigger a general mechanism of bacterial response to antimicrobial compounds due to its broad spectrum of cross-resistance against antibiotics.

These results demonstrate that emerging mutants can not only develop direct resistance against the IC applied in the evolution treatments (carvacrol in this case), but also cross-resistance to a wide range of antibiotics. Therefore, these results highlight the relevance of the genetic variations present in SeLCar for the development of AMR, which emerged through carvacrol evolution experiments but led to general antimicrobial resistance.

### 3.5. Detection of Genetic Variations in Evolved Strains

WGS was conducted on SeWT and on the evolved strains SeScar and SeLCar in order to find out which genetic variations were associated with increased resistance to carvacrol in SeSCar and SeLCar, and to antibiotics in SeLCar. A total of 3.65, 4.23 and 4.04 million of 150 bp-reads were obtained for SeWT, SeSCar and SeLCar, respectively. The average quality of the reads was 33.07, 33.05 and 33.01, and the percentage of reads above Q_30_ was 86.58 %, 86.99 % and 86.32 % for SeWT, SeSCar, and SeLCar, respectively. The quality-control-filtered paired-end reads were mapped at 98.12 %, 98.35 % and 97.94%, respectively, on the reference genome sequence (NCBI accession: NC_003197.2): *Salmonella enterica* subsp. *enterica* serovar Typhimurium str. LT2 [23]. The reference genome was sufficiently covered to allow the detection of genetic variations among the strains studied; a 150-fold coverage depth was achieved for all three strains.

The genetic variations between the reference genome and SeWT were analyzed in order to discard those mutations as the cause of the increased resistance to carvacrol in the evolved strains. In this regard, a large deletion of 1179 bp was located from 4122,950 to 4124,130 bp, and several SNVs and InDels were identified (Table S4).

**Figure 6:**
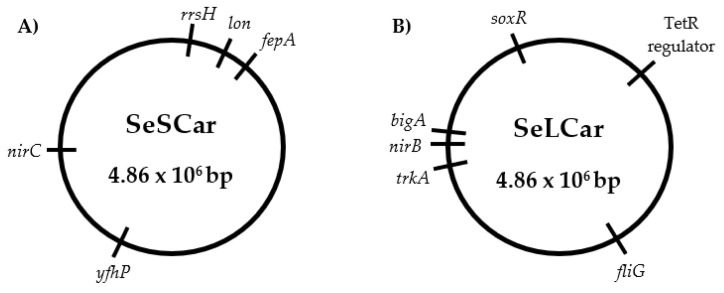
Genomic maps of the *Salmonella enterica* subsp. *enterica* serovar Typhimurium LT2 evolved strains by cyclic exposure to prolonged sublethal treatments (SeSCar; (**A**)) and to short lethal treatments (SeLCar; (**B**)) of carvacrol.

Although the sequences were mapped to the reference genome sequence, this study focused on the genetic variations between SeWT and the evolved strains (Figure 6). In this sense, knowledge of mutated genes and their relationship with the increased resistance in the evolved strains would allow us to find out the cell response mechanisms of *S.* Typhimurium against carvacrol. Genomic comparison of the strains revealed six SNVs and one insertion in SeSCar (Table 4), and five SNVs and one insertion in SeLCar (Table 5), with respect to SeWT.

#### 3.5.1. Identification of Genetic Variations in SeScar

As detailed in Table 4, the genetic variations that occurred in SeSCar via cyclic and prolonged exposure to subinhibitory doses of carvacrol were detected. In addition, the genes involved in the mutations, as well the coding proteins, were identified in order to understand the cause of the increased resistance observed.

Firstly, three SNVs were detected at positions 1121, 1130 and 1529 bp in the *rrsH* gene, a ribosomal RNA (rRNA) operon. The 16S rRNA ribosomal is the RNA component of the 30S small subunit of the ribosome and, consequently, is involved in protein synthesis. The 16S rRNA gene sequence has become the most widely used marker gene for profiling bacterial communities due to its hypervariability [41]; the mutations in SeSCar were indeed located at the variable regions 7 and 9 of the 16S rRNA. It is quite common to find mutations in this region of the genome, and they are probably not related to the increased resistance to carvacrol. 

The insertion of six nucleotides observed in the *fepA* gene, which encodes an outer membrane receptor protein involved in the uptake of enterobactin (iron siderophore), was also discarded as a cause of the increased resistance against carvacrol, since this mutation was located between the ribosome binding site (RBS) and the promoter (−10 recognition region): thus, no codifying or transcriptional regions were altered.

Another SNV was detected at position 1211 bp in the *lon* gene, causing the substitution of the amino acid glycine (Gly) by aspartic acid (Asp). The *lon* gene encodes an ATP-dependent protease (Lon proteases) that regulates the selective degradation of dysfunctional proteins and short-lived regulatory proteins. Several studies have shown that Lon protease is a stress-induced protein essential to cellular homeostasis and cell survival; it mediates protein quality control and metabolic regulation [42]. Genetic variations in *lon* would lead to a change in the efficiency of maintaining cellular homeostasis and, consequently, to an increased resistance to carvacrol. In addition, mutations in *lon* have been associated with antibiotic resistance: not directly providing intrinsic resistance, but increasing genetic instability and enhancing genetic evolution towards it [43,44]. Perhaps the genetic variation in *lon* occurred in a previous step that was necessary for the rest of the mutations involved in the bacterial response against carvacrol to occur. In addition, according to Song [45], the mutation in *lon* could play an important role in the virulence of *S.* Typhimurium: adhesion to and invasion of epithelial cells, motility and replication in macrophages.

A similar hypothesis can help to explain the SNV present in the *nirC* gene: a missense mutation was located at 215 bp, resulting in a change from valine (Val) to alanine (Ala). This gene encodes the nitrite transporter NirC, an integral membrane protein which mediates the passage of the nitrite (NO_2_^−^) and nitrate (NO_3_^−^) anions across the cytoplasmic membrane [46]. The accumulation of nitrites inside the cells could be harmful to bacteria when reduced to nitric oxide (NO), since it causes genomic alterations by deamination of the DNA [47]. Therefore, based on the function of *nirC*, this mutation does not appear to be directly responsible for the increased resistance to carvacrol in SeSCar. Perhaps an increase in enzyme efficiency could reduce the oxidative damage to bacteria induced by carvacrol. However, it is likely that if the *nirC* mutation has produced a disruption in the regulation of nitrite and nitrate anions, an increase in mutagenesis is the result. In this respect, the probability of the emergence of resistant strains in the evolution treatments would have been increased by the *nirC* mutation.

Finally, a transversion from guanine to thymine was identified in the *yfhP* gene, resulting in a change in the predicted translation from alanine (Ala) to glutamic acid (Glu). This gene regulates the transcription of several operons and genes involved in the biogenesis of Fe-S clusters and Fe-S-containing proteins. Multiple Fe-S cluster assembly pathways are present in bacteria to carry out basal, stress-responsive, and enzyme-specific cluster assembly [48]. Previous transcriptional and proteogenomic studies on *S.* Typhimurium showed a high expression of *yfhP* to chlorine treatments [49] and to hydrogen peroxide [50], suggesting its important role in cellular responses to oxidative stress. According to Chueca, Pagán and García-Gonzalo [6], carvacrol promotes endogenous generation of reactive oxygen species (ROS) and, hence, *yfhP* is probably involved in one of the response pathways to the oxidative stress caused by carvacrol. In view of the extensive literature on *yfhP* and its putative role in cellular responses to oxidative agents, this mutation is probably the main cause of increased resistance to carvacrol observed in SeSCar. However, neither this mutation nor the others observed in this strain would be related to antibiotic resistance.

#### 3.5.2. Identification of Genetic Variations in SeLcar

Genetic variations detected in the SeLcar strain, evolved under short lethal treatments of carvacrol, are summarized in Table 5. In addition, we analyzed protein coding and their functions to determine the origin of the strain’s resistance to carvacrol. 

Firstly, a SNV was detected at the position 1234 bp of the *trkA* gene, leading to a substitution of an alanine (Ala) by a threonine (Thr). This gene encodes the TrkA protein, an essential subunit of the transmembrane protein of potassium transport systems (K^+^), which plays an important role in homeostasis, in cell turgor, and in adaptation to osmotic conditions. Moreover, K+ transporters are critical to the pathogenesis of *Salmonella* in mice and chicks and are involved in multiple virulence characteristics *in vitro*, including protein secretion, motility and invasion of epithelial cells [51]. However, according to Knöppel, et al. [52], this mutation probably occurs as a result of adaptation to the laboratory growth medium, and not as a mechanism of resistance to antimicrobials. A SNV was detected at position 525 bp of the codifying region of the *bigA* gene, which results in a putative surface-exposed virulence protein BigA. Despite the close relationship observed between virulence factors and antibiotic resistance [53], this mutation was also discarded as a cause of the increased resistance, because it produced no change at the protein level (silent mutation).

In the *fliG* gene, a missense mutation was detected that produced the substitution of an asparagine (Asn) by the amino acid serine (Ser) in the position 204 aa of the FliG protein. The FliG protein forms the C-ring together with the FliN and FliM protein, a complex located at the base of the basal body of the flagellum. FliG is the most involved C-ring protein in the generation of the force necessary for flagellar mobility. Li, et al. [54] reported a down-regulation of *fliG* in *Aeromonas hydrophila* exposed to chlortetracycline. Perhaps a partial or total loss of function of *fliG* caused by the mutation could be associated with the increased resistance. However, to the best of our knowledge, no other studies have related *fliG* with AMR.

The *nirB* gene was also mutated in SeLCar at position 227 bp, resulting in the substitution of a valine (Val) by an alanine (Ala). This gene encodes the large subunit of the enzyme nitrite reductase NirBD, which transforms intracellular nitrite (NO_2_^−^) into ammonium cation (NH_4_^+^) and nitrogen (N_2_), avoiding its transformation into nitric oxide (NO) and, consequently, DNA damage [47]. As discussed above, increased enzyme efficiency could perhaps reduce oxidative damage, but if the mutation caused the alteration of this enzyme, this could lead to an increase in intracellular nitric oxide (NO), thereby resulting in a high mutation rate which would lead, in turn, to the emergence of resistant strains.

A frame shift mutation was identified by an insertion of 17 bp in the STM0580 gene, which encodes a TetR family transcriptional regulator (TFR), probably leading to a loss of protein function. TFRs are widely associated with antibiotic resistance and the regulation of genes encoding small-molecule exporters. However, TFRs play a much broader role, controlling genes involved in the metabolism, antibiotic production, quorum sensing, and many other aspects of prokaryotic physiology [55]. Abouzeed et al. [56] reported that the inactivation of this regulator resulted in an increase in the expression of *ramA* and the AcrAB efflux pump, conferring an increased resistance, not only to tetracycline, but also to a wide range of antibiotics. In addition, an evolved strain of *E. coli* also presented a SNV in *acrR*, a TFR-encoding gene, and showed an increased resistance to carvacrol and antibiotics, but in this case, it was obtained under subinhibitory doses of limonene oxide [11]. Al-Mnaser [17] also observed a mutation in a gene related to antibiotic resistance, *marR,* in a resistant *E. coli* strain isolated by subinhibitory doses, but antibiotic susceptibility was not tested. In this regard, the STM0580 gene is probably related to the AMR previously observed, and also to antibiotics and perhaps to carvacrol; however, the lack of precise information regarding this gene in *S.* Typhimurium makes it difficult to know more about its implication in the resistance against carvacrol.

Finally, a transition from cytosine to thymine was observed at position 58 bp in the *soxR* gene. Consequently, the translation would be modified from arginine to cysteine at position 20 aa, specifically in the DNA-binding-domain of the SoxR protein. This gene codes the redox-sensitive transcriptional regulator SoxR, which regulates the expression of the regulon involved in defence against redox-cycling drugs [57] and in response to nitric oxide [58]. In the presence of compounds that generate oxidative stress, the 2Fe-2S group is oxidized and acquires the capability to activate the transcription of the *soxS* gene [59]. SoxS is also a transcription factor that activates the expression of more than 100 genes of the SoxRS regulon, providing cellular defense against oxidative stress [60]. The regulon SoxRS has been extensively studied and its function in the resistance to oxidizing agents and antibiotics extensively described; however, only few studies have pointed out its important role against ICs or EOs [16,61]. The main strategy of the SoxRS regulon is to minimize intracellular drug concentration through mechanisms that impede their entry, chemically modify them, or pump them out [57]. This cellular response is likely to be activated against carvacrol, which would explain the increase in resistance of SeLCar. A missense mutation of *soxR* (Asp137Tyr) was also identified in a strain of *E. coli* evolved in the presence of subinhibitory doses of carvacrol [16]. That strain, as well as SeLCar, showed an increased resistance not only to carvacrol, but also to a wide range of antibiotics [11,16]. Koutsolioutsou, et al. [62] also identified a mutation in the soxRS regulon, providing resistance against oxidant agents and multiple antibiotics. On the one hand, these results reveal that *soxR* is a key mechanism in the cellular response to carvacrol and to several antibiotics, and supports the assumption that genetic variations of this gene may occur during evolution experiments, allowing the emergence of resistant strains. On the other hand, these data suggest that oxidative stress is strongly involved in the *Salmonella* response to carvacrol as occurs in *E. coli* [6,63], leading to an excretion of carvacrol to avoid its increase on an intracellular level. A recent proteomic study in *Salmonella* also supports that oxidative stress could be related with the cell response to carvacrol and *Origanum vulgare* EO: a differential expression of superoxide dismutase, chaperones and molecular proteases, DNA-binding protein H-NS and other stress-related proteins associated with cellular biosynthesis processes, was observed [64]. Moreover, this mutation could affect the virulence of the strain since SoxS is a positive regulator of key pathogenesis genes and promotes intracellular replication and virulence of *S.* Typhimurium [65].

In summary, both mutations identified as the main cause of increased resistance, *yfhP* in SeSCar and *soxR* in SeLCar, imply that oxidative stress might be one of the main inducers of cellular response to carvacrol. Both genes are transcriptional regulators of oxidative stress-response, the relevance of which in the defense against oxidizing agents has been previously demonstrated, as well as against antibiotics, in the case of *soxR*. In this regard, the SNVs observed in *yfhP* and *soxR* would modify the regulation of cellular response to carvacrol, resulting in increased AMR. These results highlight the likely relevance of oxidative stress-response in the cell defense to carvacrol in *Salmonella.* In addition, all the genetic variations of both strains were located in the genome, not in mobile genetics elements, such as plasmids, transposons, etc., so they would be considered hereditable mutations, and the increased resistance would be considered stable.

As described by Mao, et al. [66], evolution assays exert a selective pressure on bacteria population, which facilitates the isolation of the most resistant mutants. Those strains that show a better growth fitness in the presence of the antimicrobial agent [28] or survive lethal treatments [20] will emerge above the rest of the bacterial population. However, those mutations that occur spontaneously during bacterial growth because of replication errors can be overselected [67]; moreover, several studies support the assumption that such mutations might be induced by the treatment, even as part of the cellular response to stress [68], such as the SOS system [69]. In addition, Jee, et al. [70] and Massey and Buckling [71] argue that increased mutations would occur in specific sites or regions as an adaptive response to environmental conditions. Jinks-Robertson and Bhagwat [72] and Hudson, et al. [73] explain that mutagenesis would tend to occur in the most transcriptionally active genes during cellular response to treatments. According to these studies, AMR might not only emerge randomly and spontaneously in the course of carvacrol treatments, but the latter would also induce specific mutations provoked by the stress that improves bacterial survival. This hypothesis would support the assumption that the mutations in SeSCar and SeLCar identified herein are related to key mechanisms in the bacterial response to oxidative stress activated by carvacrol. However, depending of the mutations that occurred during the evolution assays, the behaviour of the evolved strains was different. Comparing both evolved strains, the mutations identified in the SeSCar led to a greater increase in survival against lethal carvacrol treatments, while the genetic modifications detected in the SeLCar provided an improved fitness for growth in the presence of carvacrol, as well as an increased resistance to antibiotics. Unknown phenomena of epistasis may nevertheless also occur, thereby leading to increased resistance. In addition, regardless of whether certain mutations are induced by the treatment or not, the emergence of resistant strains would be more likely and, consequently, could pose a risk to food safety that remains unexplored.

## 4. Conclusions

By cyclic exposure to prolonged sublethal treatments as well as short lethal treatments, the carvacrol evolution experiments herein described enabled the selection of strains of *S. enterica* Typhimurium that were resistant against carvacrol: SeSCar (resulting from prolonged sublethal treatments), and SeLCar (resulting from short lethal treatments). SeLCar also developed resistance to a wide range of antibiotics, such as tetracyclines, quinolones, aminoglycosides, and beta-lactams. The occurrence of stable resistance against carvacrol, which is a common constituent of many EOs recommended as food preservatives or disinfectant agents, could pose a risk to food safety. In this regard, further research is required in order to determine whether the emergence of resistant strains is dependent on the environmental conditions, the specific antimicrobial used, or it is a general phenomenon that should be considered in the design of food preservation strategies to ensure consumer health.

In this study, we adopted a novel approach to understand the antimicrobial action mechanisms of carvacrol. Whole genome sequencing (WGS) of SeSCar and SeLCar revealed the genetic variations responsible for those strains’ increased resistance to carvacrol. Considering the mutated genes that are involved in cellular defense, *yfhP* in SeSCar and *soxR* in SeLCar, we conclude that carvacrol treatments probably induce an oxidative stress response in bacteria that activates resistance mechanisms in which homeostasis plays an essential role. Furthermore, based on the mutations found, the development of resistance may be linked to variations in the virulence of *S.* Typhimurium.

While we have presented a detailed analysis suggesting the genomic causes of the observed increased resistance based on previously available data, it is certainly possible that additional genes and pathways are involved and await discovery. Therefore, further research is required to completely understand the mode of action of carvacrol on bacteria in order to enhance its antimicrobial properties as a food preservative, or as a cleaning and disinfection agent.

## Figures and Tables

**Table 1:** Minimum inhibitory concentration (MIC) and minimum bactericidal concentration (MBC) of carvacrol for *Salmonella enterica* subsp. *enterica* serovar Typhimurium LT2 wild type (SeWT) and evolved strains: SeSCar_1-5_ (5 strains selected by cyclic exposure to prolonged sublethal treatments of carvacrol) and SeLCar_1-5_ (5 strains selected by cyclic exposure to short lethal treatments of carvacrol).

Strains	MIC (µL/L)	MBC (µL/L)
**SeWT**	200	200
**SeSCar_1-5_**	300	300
**SeLCar_1-5_**	400	400

Each value represents the result of 5 different experiments carried out for every strain tested, with different bacterial cultures and on different working days.

**Table 2:** *A* (maximum OD_595_), *µ_m_* (maximum specific growth rate) and *λ* (lag time) parameters of the modified Gompertz model obtained from growth curves of *Salmonella enterica* subsp. *enterica* serovar Typhimurium LT2 wild type (SeWT) and evolved strains: SeSCar (cyclic exposure to prolonged sublethal treatments of carvacrol) and SeLCar (by cyclic exposure to short lethal treatments of carvacrol), at different concentrations of carvacrol.

***A*** **(OD_595_)**		**Strains**		
		**SeWT**	**SeSCar**	**SeLCar**
**Carvacrol (µL/L)**	**0**	0.729 ± 0.023 ^a^	0.734 ± 0.041 ^a^	0.698 ± 0.012 ^a^
	**50**	0.676 ± 0.059 ^a^	0.697 ± 0.012 ^a^	0.657 ± 0.008 ^ab^ˣ
	**100**	0.629 ± 0.051 ^a^	0.731 ± 0.026 ^a^	0.693 ± 0.046 ^a^
	**150**	0.651 ± 0.013 ^a^	0.638 ± 0.062 ^ab^	0.530 ± 0.059 ^bc^
	**200**		0.520 ± 0.026 ^b^	0.602 ± 0.020 ^abc^
	**250**		0.515 ± 0.104 ^b^	0.547 ± 0.102 ^bc^
	**300**			0.512 ± 0.041 ^c^
	**350**			0.495 ± 0.044 ^c^
***µ_m_*** **(OD_595_/h)**		**Strains**		
		**SeWT**	**SeSCar**	**SeLCar**
**Carvacrol (µL/L)**	**0**	0.480 ±0.023 ^a^	0.410 ± 0.029 ^a^	0.453 ± 0.013 ^a^
	**50**	0.325 ± 0.024 ^b^	0.351 ± 0.025 ^b^	0.367 ± 0.047 ^b^
	**100**	0.212 ± 0.022 ^c^	0.302 ± 0.006 ^bc^*	0.260 ± 0.026 ^c^
	**150**	0.024 ± 0.001 ^d^	0.262 ± 0.030 ^c^*	0.088 ± 0.023 ^d^*^†^
	**200**		0.017 ± 0.003 ^d^	0.023 ± 0.003 ^e^
	**250**		0.017 ± 0.003 ^d^	0.022 ± 0.007 ^e^
	**300**			0.017 ± 0.002 ^e^
	**350**			0.014 ±0.003 ^e^
***λ*** **(h)**		**Strains**			
		**SeWT**	**SeSCar**	**SeLCar**
**Carvacrol (µL/L)**	**0**	3.635 ± 0.104 ^a^	3.806 ± 0.078 ^a^	3.714 ± 0.069 ^a^
	**50**	3.644 ± 0.134 ^a^	3.732 ± 0.119 ^a^	3.675 ± 0.067 ^ab^
	**100**	5.189 ± 0.087 ^b^	3.748 ± 0.159 ^a^*	3.591 ± 0.057 ^a*^
	**150**	12.810 ± 0.848 ^c^	5.209 ± 0.882 ^a^*	6.051 ± 0.274 ^bc*^
	**200**		8.499 ± 1.906 ^b^	6.471 ± 0.924 ^c^
	**250**		12.253 ± 1.485 ^c^	7.494 ± 1.775 ^c^
	**300**			7.350 ± 0.167 ^c^
	**350**			12.653 ± 0.341 ^d^

Each value represents the mean ± standard deviation from 3 independent experiments. Different superscript letters represent statistically significant differences (*p* ≤ 0.05) among the means of the same column. * Significantly different from SeWT (*p* ≤ 0.05). ^†^ Significantly different from SeSCar (*p* ≤ 0.05).

**Table 3:** Zones of growth inhibition for agar disk diffusion assays of *Salmonella enterica* subsp. *enterica* serovar Typhimurium LT2 wild type (SeWT) and evolved strains: SeSCar (by cyclic exposure to prolonged sublethal treatments of carvacrol) and SeLCar (by cyclic exposure to short lethal treatments of carvacrol) against antibiotics: 30 µg kanamycin sulfate, 30 µg tetracycline, 30 µg chloramphenicol, 400 µg nalidixic acid sodium, 50 µg rifampicin, 20 µg norfloxacin, 250 µg novobiocin sodium, 10 µg trimethoprim, 10 µg ampicillin, and 150 µg cephalexin.

Antibiotics		Strains		
Antibiotic	Cell target	SeWT	SeSCar	SeLCar
**Kanamycin**	Ribosome	15.20 ± 1.40	18.65 ± 0.86	16.14 ± 1.45
**Tetracycline**	Ribosome	25.80 ± 0.80	27.96 ± 1.28	21.54 ± 1.51 *
**Chloramphenicol**	Ribosome	26.10 ± 1.37	25.19 ± 1.68	18.57 ± 0.38 *
**Nalidixic acid**	DNA synthesis	30.08 ± 1.22	34.68 ± 2.20	22.70 ± 0.81 *
**Rifampicin**	RNA synthesis	17.59 ± 0.23	17.43 ± 1.06	14.84 ± 0.52 *
**Norfloxacin**	DNA synthesis	26.43 ± 1.03	28.31 ± 1.72	20.57 ± 0.59 *
**Novobiocin**	DNA synthesis	13.63 ± 0.40	14.01 ± 0.36	9.36 ± 0.27 *
**Trimethoprim**	Thymidine synthesis pathway	27.82 ± 1.10	29.60 ± 0.90	22.81 ± 0.78 *
**Ampicillin**	Cell wall	14.12 ± 0.17	14.00 ± 0.72	9.25 ± 0.58 *
**Cephalexin**	Cell wall	22.36 ± 0.40	23.76 ± 0.97	23.73 ± 0.66

Each value represents the mean diameter of the inhibition halo ± standard deviation (mm) from three independent experiments (Ø: 6.0 mm, included). * Significantly different from SeWT (*p* ≤ 0.05).

**Table 4:** Mutations of SeSCar (strain evolved by cyclic exposure to prolonged sublethal treatments of carvacrol) in comparison with *Salmonella enterica* subsp. *enterica* serovar Typhimurium LT2 wild type (SeWT), verified by Sanger sequencing. Single nucleotide variation (SNV), insertion (Ins) and deletion (Del).

Genome Position	Gene	Locus Tag	Mutation *	Change	Information
**290,313**	*rrsH*	STM0249	SNV: G1124A	No coding	RNA 16S ribosomal
**290,319**	*rrsH*	STM0249	SNV: C1130T	No coding	RNA 16S ribosomal
**290,718**	*rrsH*	STM0249	SNV: A1529C	No coding	RNA 16S ribosomal
**506,753**	*lon*	STM0450	SNV: G1211A	Gly404Asp	Protease
**643,922**	*fepA*	STM0585	Ins: + TTTGCA 107	No coding	Membrane receptor protein
**2683,182**	*yfhP*	STM2544	SNV: C245A	Ala82Glu	HTH IscR transcriptional regulator
**3626,869**	*nirC*	STM3476	SNV: T215C	Val72Ala	Membrane transport protein (Nitrite transport)

* Position with respect to the start of the coding region.

**Table 5:** Mutations of SeLCar (strain evolved by cyclic exposure to short lethal treatments of carvacrol) in comparison with *Salmonella enterica* subsp. *enterica* serovar Typhimurium LT2 wild type (SeWT), verified by Sanger sequencing. Single nucleotide variation (SNV), insertion (Ins) and deletion (Del).

Genome Position	Gene	Locus Tag	Mutation *	Change	Information
**638,192**	-	638.192	Ins: + 17bp 522	Frame shift	TetR family transcriptional regulator
**2058,821**	*fliG*	STM1970	SNV: A611G	Asn204Ser	Flagellar protein
**3581,011**	*trkA*	STM3409	SNV: G1234A	Ala412Thr	Potassium transport regulating protein
**3623,749**	*nirB*	STM3474	SNV: T227C	Val76Ala	Large subunit nitrite reductase
**3629,699**	*bigA*	STM3478	SNV: C525T	Silent mutation (Ser175)	Putative surface-exposed virulence protein BigA
**4504,453**	*soxR*	STM4266	SNV: C58T	Arg20Cys	Redox sensitive transcriptional regulator SoxR

* Position with respect to the start of the coding region.

## Supplementary Materials

Supplementary materials can be found at https://www.mdpi.com/2076-2607/8/6/937/s1. Table S1: Primers used for PCR amplification and Sanger sequencing to verify the mutations in SeSCar and SeLCar. Table S2: Genomic background of *S.* Thypimurium LT2: a) antibiotic resistant genes and b) pathogenicity islands. Table S3: *A* (maximum OD_595_), *µ_m_* (maximum specific growth rate; h^−1^) and *λ* (lag time; h) values and error standard of the modified Gompertz models of SeWT, SeSCar and SeLCar and the goodness of the fit: *R*^2^ and adjusted *R*^2^ values and the root mean square error (*RMSE*). Table S4: Genetic variations detected by whole genome sequencing (WGS) between SeWT and the reference genome of *Salmonella enterica* subsp. *enterica* serovar Typhimurium str. LT2 (NCBI accession: NC_003197.2).
