# Supplementary material for: Genetic Variants and Phenotypic Characteristics of Salmonella Typhimurium-Resistant Mutants after Exposure to Carvacrol
Source: Microorganisms. 2020 Jun 22;8(6):937. doi: 10.3390/microorganisms8060937 (PMC7356045; doi:10.3390/microorganisms8060937)
Supplement: Supplementary file 1 [file microorganisms-08-00937-s001.pdf]

Table S1. Primers used for PCR amplification and Sanger sequencing to verify the mutations of evolved strains: SeSCar (by cyclic exposure to prolonged sublethal treatments of carvacrol) and SeLCar (by cyclic exposure to short lethal treatments of carvacrol),

SeSCar mutations	Forward primer (5′ → 3′)	Reverse primer (5′ → 3′)
rrsH	AAGAAGCACCGGCTAACTCC	CCTTTGATTCGTTTCCGGGC
lon	GCGCGTAGCAAGGTCAAAAA	ACCGTCAGTTCGCCTTTCTT
fepA	GGCATGGTGCGGATGATTTC	CGCCAGTGATGTAGACCCAG
yfhP	TTCATCCTTCAGACGACCGC	GTCAATATCGCACGGAACG
nirC	CAGCCAACAAAGAGGCAGTG	ATAAACGCCAGCAGACACCA

SeLCar mutations	Forward primer (5′ → 3′)	Reverse primer (5′ → 3′)
TetR regulator	ATCGTTGAGTAGCGAGACGG	TCGATCCATTACCGATGCCA
fliG	CAACGCCAACGAATACCTGC	GACATAGGTATCCTCGCCGC
trkA	GCGCCAAGAAAGTGATGGTG	ACCCACTGCCAAATCCACAA
nirB	AAAAGCCTGACAATTCCGCC	CGCCCATGCTTTCGATCTTG
bigA	CGCAAAACTCTCACCGACCT	GTTCAGCGTTTTACCGTCCG
soxR	CTCGGTCGTTCGTAGCTCAA	GTCTAACTCTTCGCGCCACT

Table S2. Genomic background of *S*. Thypimurium LT2: a) antibiotic resistant genes and b) pathogenicity islands.

A)

**Antibiotic resistance**			
**Resistance gene**	**Position**	**Predicted phenotype**	**Accession number**
aac(6')-Iaa	1707352..1707789	Aminoglycoside resistance	NC_003197

B)

**Pathogenic islands**			
**Gene**	**Origin**	**Position**	**Insertion location**
SPI-5	*Salmonella* Typhimurium LT2	1175309..1184377	tRNA-serT
SPI-2	*Salmonella* Typhimurium LT2	1461731..1501801	tRNA-valV
C63PI	*Salmonella* Typhimurium SL1344	2627012..2652253	fhlA
SPI-1	*Salmonella* Typhimurium LT2	3005849..3050127	fhlA-mutS
SPI-13	*Salmonella* Gallinarum SGA-10	3277144..3277484	tRNA-pheV
SPI-13	*Salmonella* Gallinarum SGG-1	3278852..3279255	tRNA-pheV
SPI-13	*Salmonella* Gallinarum SGD-3	3279563..3279900	tRNA-pheV
SPI-3	*Salmonella* Typhimurium LT2	3948588..3965203	tRNA-selC
SPI-4	*Salmonella* Choleraesuis str SC-B67	4477300..4503998	ssb-soxSR
SPI-14	*Salmonella* Gallinarum SGA-8	926728..927228	Not published
SPI-14	*Salmonella* Gallinarum SGC-8	932304..932744	Not published

Table S3. *A* (maximum OD_{595}), μ_m (maximum specific growth rate; h^{-1}) and λ (lag time; h) values and error standard of the modified Gompertz model obtained from 3 independently growth curves of *Salmonella enterica* subsp. *enterica* serovar Typhimurium LT2 wild type (SeWT) (A) and evolved strains: SeSCar (B; by cyclic exposure to prolonged sublethal treatments of carvacrol) and SeLCar (C; by cyclic exposure to short lethal treatments of carvacrol), at different concentrations of carvacrol. The goodness of the fit is shown by R^2 and adjusted R^2 values and the root mean square error (*RMSE*).

A) SeWT

**Carvacrol (μL/L)**	**Values**			**Standard error**			**Goodness of fit**		
	A	μ_{max}	λ	***A***	μ_{max}	λ	R^2	**Adj.** R^2	***RMSE***
**0**	0.7174	0.4642	3.576	0.0163	0.1491	0.248	0.9348	0.9285	0.0663
**0**	0.7565	0.4687	3.574	0.0119	0.1005	0.175	0.9680	0.9650	0.0483
**0**	0.7145	0.5069	3.755	0.0150	0.1361	0.192	0.9454	0.9402	0.0613
**50**	0.7117	0.3141	3.503	0.0239	0.1197	0.467	0.8756	0.8637	0.0940
**50**	0.7080	0.3523	3.660	0.0177	0.1014	0.313	0.9298	0.9231	0.0703
**50**	0.6070	0.3090	3.770	0.0098	0.0570	0.200	0.9701	0.9672	0.0389
**100**	0.6665	0.2116	5.279	0.0124	0.0348	0.282	0.9753	0.9730	0.0438
**100**	0.6502	0.1912	5.182	0.0133	0.0327	0.317	0.9697	0.9668	0.0463
**100**	0.5708	0.2344	5.105	0.0152	0.0663	0.379	0.9397	0.9340	0.0564
**150**	0.6429	0.0236	12.080	0.0305	0.0022	0.785	0.9719	0.9695	0.0309
**150**	0.6660	0.0242	12.610	0.0196	0.0014	0.473	0.9894	0.9885	0.0192
**150**	0.6431	0.0253	13.740	0.0314	0.0026	0.787	0.9680	0.9653	0.0321

B) SeSCar

Carvacrol (μL/L)	Values			Standard error			Godness of fit		
	A	μ_{max}	*λ*	*A*	μ_{max}	*λ*	R^2	Adj. R^2	*RMSE*
**0**	0.7584	0.4321	3.826	0.0092	0.0616	0.140	0.9832	0.9816	0.0368
**0**	0.6869	0.4195	3.873	0.0304	0.2237	0.499	0.8053	0.7868	0.1228
**0**	0.7559	0.3776	3.720	0.0171	0.0973	0.282	0.9403	0.9346	0.0678
**50**	0.6893	0.3474	3.866	0.0196	0.1144	0.366	0.9154	0.9073	0.0775
**50**	0.7099	0.3282	3.640	0.0222	0.1154	0.413	0.8954	0.8854	0.0876
**50**	0.6906	0.3787	3.691	0.0098	0.0633	0.164	0.9749	0.9725	0.0392
**100**	0.7091	0.2958	3.653	0.0062	0.0278	0.123	0.9911	0.9902	0.0242
**100**	0.7235	0.3016	3.659	0.0198	0.0883	0.383	0.9209	0.9134	0.0769
**100**	0.7601	0.3072	3.931	0.0170	0.0729	0.324	0.9486	0.9437	0.0653
**150**	0.5753	0.2336	6.227	0.0105	0.0434	0.250	0.9780	0.9759	0.0373
**150**	0.6400	0.2927	4.665	0.0134	0.0664	0.270	0.9599	0.9561	0.0512
**150**	0.6998	0.2590	4.736	0.0083	0.0300	0.171	0.9877	0.9865	0.0307
**200**	0.5247	0.0179	6.560	0.0549	0.0021	1.254	0.9132	0.9053	0.0386
**200**	0.4917	0.0142	8.567	0.0393	0.0011	0.822	0.9600	0.9564	0.0204
**200**	0.5439	0.0203	10.370	0.0293	0.0015	0.638	0.9752	0.9729	0.0204
**250**	0.4508	0.0190	13.860	0.0329	0.0024	0.862	0.9501	0.9456	0.0230
**250**	0.6351	0.0139	11.970	0.0768	0.0008	0.681	0.9712	0.9686	0.0165
**250**	0.4577	0.0179	10.930	0.0242	0.0013	0.631	0.9746	0.9723	0.0173

C) SeLCar

Carvacrol (μL/L)	Values			Standard error			Godness of fit		
	A	μ_{max}	*λ*	*A*	μ_{max}	*λ*	R^2	Adj. R^2	*RMSE*
**0**	0.6850	0.4468	3.634	0.0094	0.0826	0.142	0.9754	0.9731	0.0384
**0**	0.7017	0.4447	3.758	0.0103	0.0789	0.149	0.9736	0.9711	0.0416
**0**	0.7078	0.4684	3.750	0.0105	0.0861	0.145	0.9723	0.9696	0.0427
**50**	0.6647	0.4112	3.612	0.0161	0.1312	0.262	0.9284	0.9216	0.0654
**50**	0.6495	0.3180	3.668	0.0038	0.0213	0.074	0.9958	0.9954	0.0151
**50**	0.6570	0.3720	3.746	0.0120	0.0800	0.206	0.9598	0.9560	0.0481
**100**	0.6404	0.2889	3.612	0.0156	0.0788	0.328	0.9324	0.9259	0.0615
**100**	0.7275	0.2374	3.527	0.0064	0.0197	0.139	0.9919	0.9911	0.0241
**100**	0.7105	0.2528	3.634	0.0096	0.0338	0.205	0.9801	0.9782	0.0366
**150**	0.4898	0.0718	6.324	0.0188	0.0129	0.640	0.9462	0.9413	0.0485
**150**	0.5975	0.0789	6.054	0.0202	0.0114	0.561	0.9603	0.9567	0.0490
**150**	0.5034	0.1147	5.776	0.0104	0.0167	0.344	0.9746	0.9723	0.0342
**200**	0.6030	0.0198	5.405	0.0279	0.0010	0.558	0.9810	0.9793	0.0193
**200**	0.5818	0.0249	6.972	0.0180	0.0009	0.368	0.9908	0.9900	0.0152
**200**	0.6212	0.0254	7.036	0.0220	0.0011	0.425	0.9875	0.9863	0.0180
**250**	0.4497	0.0266	6.115	0.0297	0.0026	0.769	0.9573	0.9535	0.0320
**250**	0.6529	0.0140	9.497	0.0731	0.0009	0.725	0.9666	0.9636	0.0191
**250**	0.5375	0.0240	6.870	0.0262	0.0014	0.577	0.9769	0.9748	0.0229
**300**	0.4733	0.0188	7.425	0.0401	0.0019	1.020	0.9382	0.9326	0.0318
**300**	0.5541	0.0143	7.466	0.0435	0.0010	0.752	0.9669	0.9639	0.0197
**300**	0.5077	0.0169	7.158	0.0329	0.0012	0.765	0.9651	0.9619	0.0221
**350**	0.4467	0.0175	12.860	0.0360	0.0022	0.931	0.9463	0.9415	0.0240
**350**	0.5054	0.0136	12.840	0.0665	0.0014	0.875	0.9414	0.9361	0.0217
**350**	0.5336	0.0114	12.260	0.1156	0.0011	1.201	0.9227	0.9157	0.0228

Table S4. Genetic variations detected by whole genome sequencing (WGS) between SeWT and the reference genome of *Salmonella enterica* subsp. *enterica* serovar Typhimurium str. LT2 (NCBI accession: NC_003197.2). Single nucleotide variation (SNV), insertion (Ins) and deletion (Del).

Genome position	Genes	Locus tag	Mutation*	Change	Information
**290,718**	*rrsH*	STM0249	SNV: C1529A	No coding	RNA 16S ribosomal
**364,623**	*crl*	STM0319	Del: -T 104	Frame shift	Sigma factor-binding protein
**416,555**	*prpR*	STM0367	SNV: C1159T	Leu387Phe	Operon regulator
**453,939**	*brnQ*	STM0399	SNV: C681T	Silent mutation (Tyr227)	Branched-chain amino acid transport system carrier protein
**509,118**	*cypD*	STM0452	SNV: T450A	Asp150Glu	Peptidylprolyl isomerase
**608,859**	*fimH*	STM0547	SNV: G182C	Gly61Ala	Adhesin
**1,205,933**	Intergenic<br>*wraB - ycdF*	STM1119<br>STM1120	SNV: G →A	No coding	-
**1,778,104**	*ycjF*	STM1684	SNV: T821C	Leu274Pro	UPF0283 membrane protein
**1,841,398**	-	STM1747	SNV: G98A	Arg33Gln	Hypothetical protein
**1,849,642**	*hnr*	STM1753	SNV: T305G	Val102Gly	Regulator of RpoS
**3,469,143**	*dacB*	STM3300	SNV: C483T	Silent mutation (Ser161)	Transpeptidase
**3,673,628**	*malQ*	STM3513	SNV: T287G	Leu96Arg	4-Alpha-glucanotransferase
**3,675,952**	*malP*	STM3514	Del: -GCCGCCTG 358	Frame shift	Alpha-1,4 phosphorylase
**3,819,815**	-	STM3633	SNV: T562C	Silent mutation (Leu188)	LacI family transcriptional regulator
**4,122,937**	*gppA*	STM3913	SNV: G385T	Gly129Cys	Pyrophosphatase
**4,122,950**	*gppA*<br>*rhlB*	STM3913<br>STM3914	Del: -1,179 pb	Knock-out (*gppA*, *rhlB*)	Pyrophosphatase<br>ATP-dependent RNA helicase RhlB
**4,291,432**	*yiiQ*	STM4082	SNV: G323A	Stop-gain	Hypothetical protein
**4,294,693**	*glpK*	STM4086	SNV: G1171A	Asp391Asn	Glycerol kinase
**4,697,694**	*treB*	STM4454	Ins: + A 543	Frame shift	Pseudogene (trehalose metabolism)

*Position respect to the start of the coding region.
